# PROTOCOL: School‐based interventions for reducing disciplinary school exclusion: An updated systematic review

**DOI:** 10.1002/cl2.1344

**Published:** 2023-08-21

**Authors:** Sara Valdebenito, Hannah Gaffney, Darrick Jolliffe

**Affiliations:** ^1^ Institute of Criminology University of Cambridge Cambridge UK; ^2^ Clare Hall College University of Cambridge Cambridge UK; ^3^ Department of Law and Criminology Royal Holloway University of London London UK

## Abstract

The primary goal of the present mixed methods review is to systematically examine the available evidence for the effectiveness of different types of school‐based interventions for reducing disciplinary school exclusion. Quantitative evidence will help to understand the overall size of the impact, as well as the factors that better explain it. Qualitative evidence will help to better understand how these programmes may work, and what factors aid or hinder implementation and success.

The research questions underlying the quantitative review are as follows:

- Do school‐based programmes reduce the use of exclusionary sanctions in schools?
- Are some school‐based approaches more effective than others in reducing exclusionary sanctions?
- Do participants’ characteristics (e.g., age, sex, or ethnicity) affect the impact of school‐based programmes on exclusionary sanctions in schools?
- Do characteristics of the interventions, implementation, and methodology affect the impact of school‐based programmes on exclusionary sanctions in schools?
- Do school‐based programmes have an impact on reducing the involvement of children and young people in crime and violence?
- Do participants’ characteristics (e.g., age, gender, ethnicity) affect the impact of school‐based programmes on crime and violence?

If sufficient data are available, we will compare different approaches (e.g., school‐wide management, classroom management, restorative justice, cognitive‐behavioural interventions) and identify those that could potentially demonstrate larger effects. We will also (potentially) run analysis controlling for characteristics of *participants* (e.g., age, ethnicity, level of risk); *interventions* (e.g., theoretical bases, components); *implementation* (e.g., facilitators’ training, doses, quality); and *methodology* (e.g., research design).

The research questions underlying the qualitative review are defined as follows:

- What are the barriers and facilitators to implementation of interventions to reduce school exclusions?
- What are the barriers and facilitators to implementation of interventions to reduce the involvement of children and young people in crime and violence?

## 1 BACKGROUND

### 1.1 The problem, condition or issue

Schools are important institutions in the life of a child or young person, over and above the provision of education and academic skills (Sanders et al., 2020). A recent OECD (2021) report found that globally, from primary to secondary school, children spend around 700–800 h a year in classrooms. Apart from families, it is in schools where children are introduced to discipline, the role of authorities, the use of codes of conduct and the consequences of their transgression (Fisher et al., 2019; Maimon et al., 2012).

Resembling the justice system, schools may use a range of punitive and non‐punitive strategies to deal with transgression to codes of conduct. Among punitive responses to disciplinary problems, exclusion is regarded as one of the most serious sanctions. School exclusion, also known as suspension, is defined as a disciplinary sanction imposed by a school authority, as a consequence of student behaviour. Exclusion entails the removal of pupils from regular teaching for a period during which they are not allowed to be present in the classroom or, in more serious cases, on school premises. Under extreme circumstances, school exclusion can entail the permanent expulsion of the students, or the transference to alternative education (Valdebenito et al., 2018).

In the United Kingdom, the most recent official data suggest that for the autumn term 2021/2022 a total of 183,817 (rate of 2.21 per 1000) students were suspended or excluded from school for a fixed period and 2097 (rate of 0.03 per 1000) were permanently excluded (HM Government, 2022). These figures represent an increase from the numbers of exclusions in English schools for the previous year but remain lower than the rate of exclusions in pre‐pandemic years (HM Government, 2022).

There are several reasons for why a student may be excluded from school. In England, ‘persistent disruptive behaviour’ was noted as the reason for 41% of all fixed‐term exclusions during autumn term 2021/2022 and 31% of all permanent exclusions in the same timeframe. The types of behaviours exhibited by students who were excluded were serious and are cause for concern. For example, 16% of students who were permanently excluded from school in England during the autumn term 2021/2022 were excluded because they physically assaulted another student. A further 13% and 12.5%, respectively were excluded for verbal abuse or threatening behaviour and physical assault against an adult (HM Government, 2022).

Research has consistently found that school exclusion is linked to severe negative life outcomes for children in at least three different dimensions: behaviour, academic performance and future social inclusion. Referring to the behavioural outcomes Cohen et al. (2023), using a sample of predominantly Black school children found that in‐school and out‐of‐school exclusion were both linked with less prosocial behaviour, lower emotional regulation, an increase in disruptive behaviour and concentration problems. Arnez and Condry (2021) have discussed the concept of the ‘school‐to‐prison pipeline’ referring to the observation that children who are excluded from school seem more likely to engage in criminal, ant‐isocial, and/or delinquent behaviour ending in their imprisonment (McAra & McVie, 2010).

In the academic dimension, being excluded from school in early childhood has been associated with poorer academic outcomes as the child progresses through education (Andrew & Blake, 2023). What is more, Leban and Masterson (2022) found that school suspension by age 12 increased the chances of a student dropping out of school even after controlling for ethnicity, high exposure to adverse childhood experiences and other demographic and behavioural covariates. In line with these findings, Chu and Ready (2018) suggested that in the long term, students who have been excluded at grade 9 were more likely to drop out and less likely to graduate from high school during the following 4, 5 or 6 years when compared with non‐excluded pupils.

Finally, as suggested by Madia et al. (2022), being excluded from school increased the likelihood that a young person would be socially excluded and categorised as ‘NEET’ (Not in Education, Employment or Training) at 19–20 years old and be unemployed and on lower wages at age 25–26 years old. Also, Kupchik and Catlaw (2015) evaluated the long‐term impact of school exclusion on future political and civic participation. In particular, the study found that suspended pupils were less likely to vote in subsequent years, suggesting that suspensions had a negative effect on political participation.

The decision to exclude a child from school, either permanently or temporarily, may be necessary, but involves a series of correlated consequences and difficult decisions for school authorities. In Edward Timpson's review of school exclusion practices in England, the authors assert that:

Schools must be calm and safe places, and it is right that we fully support head teachers in using exclusion where this is appropriate. Head teachers considering exclusion have a tough choice to make, having to weigh the profound implications that it can have on a young person's life with the interests and needs of pupils and staff in the wider school community. (Timpson, 2019, p. 3)

Therefore, interventions that can effectively reduce the *need* for excluding children from schools can benefit the whole school community as well as the individual child at risk of exclusion. Furthermore, interventions to reduce the need for school exclusions also need to address the overrepresentation of certain groups among students who are excluded. In England, students who are male, belong to certain minority ethnic groups, are eligible for free school meals and have a special educational need have been historically over‐represented among students who are excluded from school, either permanently or for a fixed period (HM Government, 2022). Racial and ethnic disproportionality in the use of school exclusions (Demie, 2021; Skiba et al., 2014; Towl & Hemphill, 2016) may act as a mechanism that reinforces and maintains the overrepresentation of minorities in disadvantaged groups (e.g., socioeconomic status or those in the criminal justice system). Moreover, groups in society that are more vulnerable are also more likely to be excluded from school, for example, children with an impairing psychopathology or special educational needs (Parker et al., 2015).

### 1.2 The intervention

Throughout this protocol we refer to ‘school exclusions’, a term which in the broadest conceptualisation reflects the removal of a child from their school. This can be a temporary situation whereby a child is removed from normal school activities for a fixed period (i.e., suspension or fixed‐term exclusion) but returns following exclusion. This type of exclusion may take place either in school buildings, where a student is confined to a designated room (i.e., in‐school suspension) or where a student remains at home during the period of exclusion (i.e., out‐of‐school suspension). School exclusion can also refer to permanent exclusion, where a child is removed from the school community and is not permitted to return. For consistency, we use the phrase ‘school exclusion’ to refer to both temporary and permanent exclusions in this protocol, unless otherwise specified (HM Government, 2022).

School exclusion as a disciplinary measure could be described as a form of intervention itself. School leaders or head teachers may use exclusions as an intervention or prevention strategy for disruptive behaviour among students. However, the estimation of a ‘treatment effect’ for school exclusion is understudied (Sutherland & Eisner, 2014). The efficacy of exclusions is also unfounded. Theriot et al. (2010) identified that students who received either in‐school suspension or out‐of‐school suspension were more likely to be excluded again.

In the UK policy context, other available options include ‘managed moves’, whereby a child who is at risk of exclusion from one school may be moved to another or moved to a pupil referral unit/alternate provision (Thomson, 2019). ‘Off rolling’ is another way in which schools may deal with children who are at risk of permanent exclusion without using official channels (Timpson, 2019). Parents of a child at risk of exclusion may be pressured into removing their child from one school before an exclusion takes place, and while this is not necessarily illegal, official bodies have deemed it unacceptable practice (Owen, 2019).

However, there are a number of intervention and prevention strategies that schools can employ before a child is considered for exclusion from school. These interventions are the focus of our proposed systematic review and meta‐analysis. These interventions are often multifaceted and multidisciplinary. In the previous review Valdebenito et al. (2018), the disciplinary background of authors of evaluations was examined. The majority of authors were labelled as having a background in education (35.3%) or psychology (32.3%) unsurprisingly, other discipline backgrounds included social work, criminal justice, psychiatry or medicine, and econometrics or economics (Valdebenito et al., 2018).

Thus, in our updated review, we propose to include all school‐based interventions that report an impact on exclusion or suspension outcomes. This will encompass interventions implemented at every educational stage, from primary school through to secondary schools (or high schools in some locations). We also propose to include multi‐system interventions with the only caveat being that at least one element of the intervention had to be implemented in a school setting. As a result, there are many different types of interventions that may be included in our review, for example, school‐based mental health interventions that involve implementing one‐to‐one counselling (Toth et al., 2022) or interventions to create more equitable discipline policies in schools (Gregory & Skiba, 2019).

We expect that most interventions that will be included in the present review will target behavioural change among children at risk of being excluded or the wider student population. We anticipate that there will be a variety of single‐component (i.e., those that target individual‐ or school‐level risk factors) and complex multi‐component or ‘whole school’ intervention programmes. In the previous review, Valdebenito et al. (2018) identified a range of different interventions that had been evaluated using randomised controlled trials to reduce school exclusions. Table 1 below summarises the types of interventions included in this earlier publication.

**Table 1:** Types of intervention programmes.

Programme type	Number of studies	% of studies (%)
Enhancement of academic skills	2	5.4
After‐school programmes	2	5.4
Mentoring/monitoring	5	13.5
Skills training for students	9	24.3
Skills training for teachers	3	8.1
School‐wide strategies	6	16.2
Violence reduction	3	8.1
Counselling, mental health	3	8.1
Other	4	10.8

*Source*: Adapted from Valdebenito et al. (2018, p. 61).

We have not altered the inclusion criteria for the proposed updated systematic review and meta‐analysis with respect to the types of interventions, and therefore, expect that similar types of interventions will be identified.

Given the research, policy, and practice attention that the concept of school exclusions has received in recent years, we expect that in this updated review we may also identify evaluations of the effectiveness of policy changes either within schools or on a national/state level. For instance, zero tolerance policies, Behavioural Threat Assessment and uniform policies (e.g., Draa, 2005; Gentile & Imberman, 2012; Gouge, 2011; Johnson, 2010; Samuels & Bishop, 2003; Shimizu & Peterson, 2000; Stevenson & Brooks, 1999; Vaughan, 2001; Washington‐Labat & Ginn, 2003). There are challenges in evaluating the impact of overarching policies because it is not always clear when these started, the extent to which these policies have been implemented (dosage), and the challenge of identifying an adequate counterfactual. Such evaluations will be considered for inclusion in our proposed review provided they meet other inclusion criteria.

### 1.3 How the intervention might work

Previous research has indicated the need for intervention programmes that address both student behaviour and the school environment to reduce the number of students that are excluded (Theriot et al., 2010). Therefore, the theories of change underlying interventions to reduce school exclusions are likely to focus on individual‐ or school‐level change, or both. As such, interventions to prevent or reduce the frequency of school exclusions may work through various mechanisms. These mechanisms will depend on the type of intervention programme implemented.

Valdebenito et al. (2018) noted that theoretical foundations were not commonly reported in the primary evaluations of interventions to reduce school exclusions. It was more common for evaluations to report a set of intervention components or activities that were implemented. Valdebenito et al. (2018) categorised interventions using this limited information and reported that the majority were based on a cognitive behavioural framework or an ecological systems theory.

On the system or school‐level, interventions may aim to reduce school exclusions by targeting the ‘school climate’. This possible mechanism of change involves creating a school climate that promotes supportive relationships and encourages positive behaviour (Scottish Government, 2017).

At the individual‐level, interventions may aim to directly change students' behaviour, particularly disruptive, violent, or aggressive behaviour, to reduce the risk of a child being excluded from school. Interventions may also target risk factors for exclusion such as truancy (Keppens & Spruyt, 2020) or specific behaviours directly, for example using anger management or cognitive behavioural therapy techniques (Feindler & Engel, 2011).

### 1.4 Why it is important to do this review

There are several reasons that justify the present review. First, the significant relationship between school exclusions and several negative life outcomes, such as the involvement in crime and violence (Gerlinger et al., 2021; Novak, 2019). Much research has shown that being excluded from school is a ‘critical moment’ in a young person's development and is an important factor that can hinder desistance from offending (McAra & McVie, 2010).

Many researchers have explored the phenomenon referred to as the ‘school‐to‐prison pipeline’, which is the finding that those who are excluded from school are at in increased risk of later custodial sentences. Recently, using national data from England, Cathro et al. (2023) found that among all youth who were serving a custodial sentence and aged 15–17 years old, almost 50% had been excluded from school at least once. Similarly, in New Zealand, Sanders et al. (2020) assessed the relationship between being excluded from school and criminal justice system involvement. The former was measured as the sum of three items related to being ‘stood down’ (i.e., when a student is told to not come to school the day after they are sent home; Sanders et al., 2020), fixed‐term exclusions and permanent exclusions. Criminal justice involvement included indicators of whether a young person had been arrested, gone to court, or received a custodial sentence in the previous 12 months (Sanders et al., 2020). The results indicated that there was both a direct relationship between exclusion and criminal justice involvement and an indirect relationship through delinquency (e.g., theft, vandalism, and aggressive behaviour; Sanders et al., 2020).

Therefore, it is important to undertake this mixed‐methods review to better our understanding of the effectiveness of interventions to reduce school exclusions. By reducing the number of children who are excluded from school, one may reduce the number of children involved in crime and violence. This is something we plan to explore in the proposed updated systematic review and meta‐analysis.

Second, as previously discussed, this protocol refers to an update of an earlier Campbell systematic review and meta‐analysis of interventions to reduce school exclusions (Valdebenito et al., 2018). The searches for this review were completed in 2015 and this review was the first attempt to synthesise the effectiveness of interventions to reduce school exclusions. Thus, another reason to undertake the proposed update is to further our understanding of what works to reduce the number of students who are excluded from school by including the most recent research. The proposed mixed‐methods systematic review and meta‐analysis will also represent a significant contribution to the literature in this area. The previous review included only impact evaluations using a randomised controlled trial design (Valdebenito et al., 2018) but our proposed update will also aim to include high quality quasi‐experimental designs and process evaluations to better our understanding of the effectiveness and implementation of interventions to reduce school exclusions.

#### 1.4.1 Previous reviews and meta‐analyses

Given the later negative outcomes associated with school exclusion, it is surprising that there remains little research evidence on intervention efficacy in this area. We identified two recent and relevant systematic reviews (Gage et al., 2018; Mielke & Farrington, 2021). The most relevant systematic review and meta‐analysis focused only on interventions to reduce suspensions (i.e., fixed‐term exclusions) and arrests (Mielke & Farrington, 2021). The results showed that, overall, interventions were effective in reducing suspensions and arrests, but the weighted mean effect sizes were not statistically significant and only 14 studies were included (Mielke & Farrington, 2021). This review was the first to synthesise the impact of school‐based intervention programmes to reduce suspensions and offending outcomes. The review by Gage et al. (2018) focussed only on one specific intervention—Schoolwide Positive Behaviour Interventions and Supports (SWPBIS). Despite this intervention being widely implemented (approximately 23,000 schools; Gage et al., 2018) only four experimental or quasi‐experimental evaluations were identified.

## 2 OBJECTIVES

The primary goal of the present mixed methods review is to systematically examine the available evidence for the effectiveness of different types of school‐based interventions for reducing disciplinary school exclusion. Quantitative evidence will help to understand the overall size of the impact, as well as the factors that better explain it. Qualitative evidence will help to better understand how these programmes may work, and what factors aid or hinder implementation and success.

The research questions underlying the quantitative review are as follows:

- Do school‐based programmes reduce the use of exclusionary sanctions in schools?
- Are some school‐based approaches more effective than others in reducing exclusionary sanctions?
- Do participants' characteristics (e.g., age, sex, or ethnicity) affect the impact of school‐based programmes on exclusionary sanctions in schools?
- Do characteristics of the interventions, implementation, and methodology affect the impact of school‐based programmes on exclusionary sanctions in schools?
- Do school‐based programmes have an impact on reducing the involvement of children and young people in crime and violence?
- Do participants' characteristics (e.g., age, gender, ethnicity) affect the impact of school‐based programmes on crime and violence?

If sufficient data are available, we will compare different approaches (e.g., school‐wide management, classroom management, restorative justice, cognitive‐behavioural interventions) and identify those that could potentially demonstrate larger effects. We will also (potentially) run analysis controlling for characteristics of *participants* (e.g., age, ethnicity, level of risk); *interventions* (e.g., theoretical bases, components); *implementation* (e.g., facilitators' training, doses, quality); and *methodology* (e.g., research design).

The research questions underlying the qualitative review are defined as follows:

- What are the barriers and facilitators to implementation of interventions to reduce school exclusions?
- What are the barriers and facilitators to implementation of interventions to reduce the involvement of children and young people in crime and violence?

## 3 METHODOLOGY

### 3.1 Criteria for considering studies for this review

#### 3.1.1 Types of studies

We will include studies based on experimental quasi‐experimental and qualitative designs.

To be included, experimental studies or randomised controlled trials (RCT) should involve at least one experimental group (i.e., participants receiving the treatment) and one control group (e.g., comparison group) with participants being randomly allocated to each condition.

The control condition in the experimental studies may involve, for instance, a control group with no intervention, a control group with intervention as usual, a wait‐list control group or a placebo group. Trials involving clustered samples will also be included. However, we plan to correct the combination of individually and clustered data (see Section 3.3.2).

Included quasi‐experimental designs (QED) should use both control group and pre/posttest. To be included, the treatment and control group should be selected in a way that the effect of selection bias is statistically controlled. The design should report clearly the method used to ensure statistical equivalence (e.g., propensity score matching, matching through cohort controls), taking into account for instance behavioural risk factors and demographic characteristics. Studies where there is a large difference between the treatment and control group at pre‐test will be excluded as they will not help in distinguishing intervention effects from other effects (Piquero et al., 2008; Shadish et al., 2002).

Quasi‐experimental studies based on one‐group pre‐test/post‐test design, repeated measures panel designs, or the one‐group posttest‐only designs will be excluded from the present review.

Since we plan to code effect size from primary studies indicating whether they have been adjusted for other covariates, when data allow, we will report adjusted and unadjusted effects in the final review separately.

We will also search and retrieve the most complete collection of primary qualitative findings that fit the aim of the present review and the inclusion/exclusion criteria. Qualitative evidence may include but not be restricted to ethnographic studies, phenomenological studies, grounded theory research or case studies involving the UK population.

In the final review, analysis and results based on RCT and QED and qualitative studies will be reported separately.

#### 3.1.2 Types of participants

Included reports should sample a general population of students in primary and secondary schools irrespective of nationality, language, and cultural or socio‐economical background. Samples from countries other than the United Kingdom will be included as long as they represent equivalent school levels.

By targeting primary and secondary schools, the sample will consist of children aged approximately 4–18.^1^ However, we expect the bulk of studies to be targeting pupils aged about 10–15, where research suggests the largest number of exclusions takes place (e.g., Liu, 2013; Raush & Skiba, 2004).

Reports involving students who present special education needs, disabilities or learning problems but settled in mainstream schools will also be included.

Reports involving students with serious mental disabilities or those in need of special schools will not be included. The rationale for this decision rests in the idea that for the present review, pupils included should represent a general population of students.

Students in college or upper levels of education will be excluded from our review.

#### 3.1.3 Types of interventions

We understand as school‐based all the interventions delivered in schools, supported by schools, or which have at least one intervention component implemented in the school setting.

In the present review we will target school‐based interventions aimed at reducing school exclusion or at least measuring exclusion rates as an outcome. Interventions can cover a wide range of psychosocial strategies and target individuals (e.g., students, teachers) or the whole school community (e.g., school‐wide positive behavioural interventions and supports [SWPBIS]). Types of intervention can include, for instance, those focussed on instructing students to identify risky behaviours and expanding their alternatives for responding appropriately to risks or harms (e.g., life skills training); interventions focussing on managing classrooms (e.g., rewards schemes), cognitive‐behavioural treatment (e.g., anger management), counselling and social work, and mentoring programmes; interventions inspired by restorative justice principles (e.g., peer mediation, restorative conferences, restorative circles); and interventions targeting teachers' skills to improve the quality of their management in the classroom (see examples on Supporting Information: Appendix 2). Programmes combining some of these strategies will also be included, as in the systematic review developed by Wilson et al. (2001).

We will exclude studies where the intervention is not school‐based or school‐supported (i.e., at least one component of the intervention should be implemented in school or by school staff). For instance, we will exclude community programmes or mental health interventions without any connection to schools.

We plan to exclude interventions designed for children or adolescents who have committed a crime, namely specialised interventions aimed at reducing reoffending or reconviction (e.g., reasoning and rehabilitation). Those interventions will be excluded because they exceed the strategies used by schools to prevent exclusion and their levels of specialisation make them not a priority for a general population of students. We will also exclude school‐based prevention programmes targeting outcomes related only to students' physical health (e.g., AIDS/HIV prevention programmes, prevention of pregnancy, programmes to develop healthy nutrition).

Different types of interventions will not be synthesised. We anticipate that interventions targeting individuals will be analysed independently of those more comprehensive in nature (i.e., school wide approaches).

#### 3.1.4 Types of outcome measures: Impact evaluations

Studies will be eligible for inclusion if they address school exclusion as an outcome. As mentioned above, school exclusion is defined as an official disciplinary sanction imposed by an authority and consisting of the removal of a child from their normal schooling. This removal should happen as a reaction to student behaviour that violates the school rules or is illegal. School exclusion can be fixed or permanent depending on the country and it can be implemented on or off school premises. All these different types of exclusion will be included in this review. We will use a range of possible search terms for exclusion that incorporate different languages and terminology from several jurisdictions. In general, all of them will target one or both of the following disciplinary sanctions: (i) fixed‐term exclusions (e.g., in‐school or out‐of‐school), and (ii) permanent exclusions (i.e., expulsion). Analysis of these two different outcomes will be carried out independently.

We will exclude other disciplinary sanctions implemented in schools if they do not share the criteria described above. For instance, we will exclude disciplinary sanctions such as loss of privileges, extra work, break/lunch detention, and after‐school detentions. They do not imply exclusion from school or exclusion from regular teaching hours and in that sense, they are not covered by this review.

For any identified study that reports findings on school exclusion as an outcome, we will also code effects of the intervention on additional outcomes that may be reported in primary studies. We are particularly interested in extracting effect sizes for offending outcomes and for outcomes that are risk factors for offending (e.g., aggression, externalising behaviour, gang involvement, impulsivity, self‐control, academic achievement, bullying etc).

#### 3.1.5 Timeframe

The present review will update the results published by Valdebenito et al. (2018). As such, databases and journals will be searched from October 2015 onwards with the aim of including contemporary interventions or prevention programmes not captured by the first version of the review.

#### 3.1.6 Publications

To be eligible, studies can be either published or unpublished reports. Sources included would be book chapters, journal articles, government reports, and also academic MSc and PhD theses. Additionally, it is important to mention that when needed, some information would be obtained through email communication with the authors or researchers in charge of a given study (e.g., statistical results).

#### 3.1.7 Language

Eligible studies can come from any country or be written in any language as long as the title, abstract and key words are written in English. The inclusion of non‐English studies will also be contingent upon resources and availability of translation services.

We have developed inclusion/exclusion screening tools that will help the members of the team to screen each manuscript (see Supporting Information: Appendix 3).

### 3.2 Search strategy

The proposed review will intend to locate and retrieve the most complete collection of empirical studies (e.g., from different countries and databases, published or unpublished). A great effort will be made to implement an exhaustive search, capable of reducing potential publication bias that could influence overall effect sizes. All searches will be conducted using a selected set of keywords. The latter will cover four main dimensions: type of study, type of interventions, population and outcomes. Table 2 describes the proposed key words for searching in the four dimensions.

**Table 2:** Proposed key words.

Type of study	Interventions	Population	Outcomes
Evaluation Effectiveness Intervention Programme Programme effectiveness Impact Effect Experimental evaluation Quasi‐experimental evaluation RCT Random evaluation Efficacy trial Process evaluation Implementation Facilitators Access Barriers	Disciplinary methods Token economy Classroom management programme/intervention/strategies School management Early interventions School support projects Skills training	Schoolchildren Pupils Children Adolescents School‐aged children Student Youth Adolescent Young people	School exclusion School exclusion reduction Suspension reduction Out‐of‐school suspension In‐school suspension Out‐of‐school exclusion In‐school exclusion Suspended Suspension Expelled Expulsion Outdoor suspension Stand‐down Exclusionary discipline Discipline

The above‐mentioned key words will be combined using Boolean operators (e.g., AND, OR, NOT), wildcards and truncation symbols with the aim of running effective searches. Since different electronic databases accept different symbols, we will create specific combinations of terms, using key words and symbols as appropriate. We will keep a precise record of each search, including for instance the key words used, their combination, the date the search is performed, the sources consulted to identify eligible studies (e.g., electronic databases, list of references, hand searches), the total number of studies located, and total number of studies retrieved. Following (Kugley et al., 2017) when possible, we will attempt to adapt searches using subject headings or descriptors appropriate for the topic and data base (for databases specialised in education, such as ERIC and CBCA Canada, we will use thesaurus or subject index).

We will use the electronic software Endnote for administering all relevant bibliographic references.

#### 3.2.1 Electronic searches of bibliographic databases

The following list details the electronic databases to be searched involving published (e.g., ISI web of knowledge, PsycINFO) and unpublished reports (e.g., Dissertation Abstracts) as well as reports in languages other than English (e.g., Scientific Electronic Library Online – SciELO).

Published papers:

– Australian Education Index (AEI)
– British Education Index (BEI) via EBSCO
– The Campbell Collaboration Social, Psychological, Educational and Criminological Trials Register (C2‐SPECTR)
– CBCA Education (Canada)
– Criminal Justice Abstracts via EBSCO
– Database of Abstracts of Reviews of Effects (DARE)
– Educational Resources Information Center (ERIC) via EBSCO
– EMBASE via OVID
– Institute of Education Sciences—What Works Clearinghouse (https://ies.ed.gov/ncee/wwc/)
– ISI Web of Science via Clarivate
– MEDLINE via PubMed
– The National Dropout Prevention Center/Network
– APA PsychInfo via EBSCO
– Sociological Abstracts via ProQuest
– Social Sciences Citation Index (SSCI) via ISI Web of Science
– Scientific Electronic Library Online (SciELO). Electronic database collecting scientific production from Latin America and the Caribbean (https://scielo.org/) via ISI Web of Science
– World Health Organisation International Clinical Trials Registry (WHO ICTRP)

Unpublished theses and dissertations:

– Dissertation Abstracts via ProQuest
– EThOS and theses
  Trial registries
– BMJ controlled trials.
– ClinicalTrial.gov
– Cochrane Central Register of Controlled Trials (CENTRAL)

Google and google scholar:

– We will complement our searches using google and google scholar. It will facilitate the access to technical reports or governmental publications. To supplement our search, we will conduct forward citation searches for each paper that was included in Valdebenito et al. (2018) using Google Scholar. In addition, we will perform backward citation searching for references cited in both primary studies and reviews related to the topic that were identified during our current search.

For each database we will run pilot searches including the key terms depicted in Table 2. They will help to adjust the terms, synonyms, and wildcards as appropriate. The pilot searches will also be helpful in creating combinations of terms that will capture relevant sets of studies. Some examples of these combinations are stated in Supporting Information: Appendix 4.

To produce a transparent report of the methodological decisions, we will keep a record of the electronic searches (e.g., date of searches, number of reports found, retrieved, key terms included, synonyms and wildcards used when appropriate). We plan to generate electronic alerts to be aware of the most recent publications in the field published during the whole process timetable of the present review.

If our initial searches are older than 12 months at the time of final publication, we will re‐run searches to publish an updated review.

#### 3.2.2 Contacting key authors

We plan to identify and contact key authors requesting information on primary studies that could be potentially integrated in this systematic review and meta‐analysis.

Additionally, in the event that papers found do not offer sufficient statistical data, the main authors will be contacted with a request for more detail.

#### 3.2.3 List of references

We propose to review reference lists of previous primary studies (e.g.,) or reviews related to the intervention/outcomes (e.g., Mielke & Farrington, 2021). Previous experiences demonstrate that this exercise produces an extra stock of manuscripts (e.g., Farrington & Ttofi, 2010).

We will also conduct citation searches using Google Scholar to screen studies that have referenced evaluations included in the previous systematic review and meta‐analysis.

#### 3.2.4 Hand searches

We will hand‐search journals specialised in education research or evaluation research if they are not available online. We will run hand searches looking at the top five journals in our list of included studies. We will look at tables of content and special issues from October 2015.

##### Websites of national and international organisation

Our search will involve exploring the websites of both national and international organisations that have generated evidence on the topic of education. The following list describe the selected resources:

www.unicef.org/education

www.unesco.org/en/publications

www.worldbank.org/en/research/brief/publications

www.education.ec.europa.eu/

www.idrc.ca/en/program/education-and-science

www.caf.com/en/topics/e/education/

educationendowmentfoundation.org.uk/

excludedlives.education.ox.ac.uk/excluded-lives-project/

www.civilrightsproject.ucla.edu/research

www.togethertransformingbehaviour.com/

www.ippr.org/

We have chosen these agencies based on recommendations from experts and our prior familiarity with the work performed by them.

### 3.3 Description of methods used in primary impact evaluations

#### 3.3.1 Type of studies

In the field of education research, the number of experimental studies is limited. For many reasons, these types of studies are not always feasible: Large‐scale trials are expensive and demanding, the availability of the sample is sometimes restricted, there are methodological difficulties in randomising school populations (individuals vs. clusters), and there are some ethical concerns about the children in the control group who are not receiving treatment although they could potentially need it. We hope to find the most reliable randomised controlled trials, but knowing that they could be a minority, quasi‐experiments will also be included. Studies using a quasi‐experimental design should involve pre‐ and post‐intervention measures as well as a control condition. More details have been provided in the sub heading types of study design.

We plan to run moderator analysis controlling by the type of designs implemented in the primary sources. Issues with study design will also be captured in our planned risk of bias assessment (see Section 3.6).

#### 3.3.2 Unit of analysis

For the purposes of this systematic review, we plan to include primary studies involving pupils and clusters of pupils as units of analysis. One key issue emerges when meta‐analysis includes studies randomising clusters or units. Participants nested in the same cluster tend to share similarities (intra‐cluster correlation [ICC]). When this correlation is not accounted for, standard errors, confidence intervals and *p*‐values will tend to be too small. These conditions affect the meta‐analysis in two different ways. First, the primary trial gets a mistakenly high weight. Second, the pooled result produces a meta‐analysis with an overly small standard error. To avoid the combination of individual and clustered data we plan to follow the strategy proposed by the *Cochrane Handbook for Systematic Reviews of Interventions* (Higgins & Green, 2011). The handbook suggests that the effective sample size in a cluster‐randomised trial can be obtained dividing the original sample size by the *design effect*, which equals 1 + (*M* − 1) × ICC. In this equation, *M* is the average cluster size and ICC is the intra‐cluster correlation coefficient.

### 3.4 Criteria for determination of independent findings

As previously mentioned, the sources included in this review will be book chapters, journal articles, government reports, and academic MSc and PhD theses. In some cases, the same data would be published in more than one source (e.g., a book chapter and a journal article). To avoid the overestimation of the effect sizes, data will be coded just once. Following Lipsey and Landenberger (2006), in the above‐mentioned cases we will code the most frequent result across the set of sources. In cases where this criterion is not enough, we will choose the most complete record of the evaluation.

We will deal with dependence using robust variance estimation (RVE) and an appropriate working model for meta‐analysis with dependent effects. We expect that effect sizes will be dependent within studies and also between studies, therefore, a correlated hierarchical working model (Pustejovsky & Tipton, 2022) is likely to be the most appropriate approach.

### 3.5 Details of study coding categories

#### 3.5.1 Coded variables

For the purposes of this meta‐analysis, studies will be coded in terms of *publication features* (e.g., author, year of publication, language), *methodology* (e.g., research design, sampling methods, attrition), *participants* (e.g., age, ethnicity, gender), *characteristics of the intervention* (e.g., setting, doses, training), *role of the evaluator* (e.g., dependent, independent evaluator), and the *outcomes measured* (e.g., school exclusion). Supporting Information: Appendix 1 offers a detailed scheme of the variables to be codified. In relation to information about participants that will be coded we plan to use labels for ethnicity and gender, or biological sex and gender‐identity, that are reported in primary studies.

#### 3.5.2 Coding process and coding reliability

The process for screening studies for inclusion/exclusion will be organised in two stages. First, we will identify our targeted studies based on titles, abstracts and key words. The second stage of the screening will be based on the reading of the full text, including any relevant retraction statements and errata notes. Retraction statements and errata would be important for assessing study limitations or study quality (Higgins & Green, 2011).

Two trained coders (i.e., one of the authors and a trained research assistant) will work independently on deciding the inclusion/exclusion of reports following the predefined criteria in Section 3.1. The participation of two independent coders is aimed at reducing bias and reducing the risk of making mistakes.

Coders will be in charge of extracting data from each included study using the data collection instrument in Supporting Information: Appendix 1. This information will be entered into an electronic database to produce descriptive/inferential statistics. In the event of discrepancies between coders at any stage of the process, the principal investigator will take part in the decision‐making process. Discrepancies will be solved by consensus. We will keep a record of discrepancies, involving the independent coding plus the final agreement.

The procedures for searching manuscripts as well as the screening for each manuscript's inclusion or exclusion will be documented in detail. In the final review we will use these details to produce a PRISMA flow chart (Liberati, 2009).

### 3.6 Statistical procedures and conventions

#### 3.6.1 Effect size metrics

Effect sizes will be computed using Borenstein et al. (2009) as well as Lipsey and Wilson (2001). If necessary, effect sizes may be computed using the Campbell Collaboration effect size calculator or in consultation with experts.

Since measures of school exclusion are mainly expressed in raw frequencies of exclusion, percentages, proportions and rates (e.g., number of days suspended/excluded from school divided by 100 students), we will use odds ratios (ORs) as the main metric for the primary outcome. Consequently, our results will express the ratio of the odds of being excluded from school (event) for those in the treatment and control groups. ORs computation will be carried out on a natural log scale with the purpose of maintaining symmetry in the analysis. Log OR and the standard error of the log will then be converted back to original OR metric to facilitate substantive interpretation, as advised by Borenstein et al. (2009). In all the cases, ORs will be reported along with 95% confidence intervals.

Where an OR is the desired effect size, our review will use the following rules to guide interpretation. An OR greater than 1 will represent a desirable intervention effect, or a reduction in school exclusions or an increase in positive outcomes (e.g., academic attainment). An OR that equals 1 will represent a null intervention effect, and an OR less than 1 will represent an undesirable intervention effect (e.g., an increase in school exclusions or a decrease in academic attainment). Where applicable, the direction of effect sizes may need to be adjusted (i.e., multiplied by −1) so that all effects are coded in the same direction.

Outcomes could also be reported using continuous scales. If sufficient data is reported we will calculate standardised mean difference (SMD) or Cohen's *d* as the main metric for such outcomes. In the event of a small sample size, SMD will be corrected by transforming the point estimate into Hedges' *g*, using the formula in Lipsey and Wilson (2001). The estimated parameters will be reported along with 95% confidence intervals.

On the assumption that we find a mixture of both binary and continuous data for our targeted outcomes, we will save the original metric in the data collection instrument and transform the less frequent effect size into the more common metric for a given outcome. In the case of results expressed in raw data along with log‐transformed data we will proceed to transformation following specialised criteria and formulas such as those proposed by Higgins and Green (2011) in section 9.4 of the *Cochrane Handbook for Systematic Reviews of Interventions*. We will run sensitivity analysis for any differences caused by potential transformation.

#### 3.6.2 Dependent effect sizes

We plan to extract as many relevant effect sizes as possible from included primary studies. This may be where studies report the effect on multiple outcomes (e.g., suspension and exclusion from school) or for multiple waves of data collection (e.g., immediate post‐intervention and 1 year follow‐up). Where possible we will also aim to extract effect sizes for subgroups in primary studies, for example, when the impact of the intervention is estimated for males and females or different ethnic groups separately.

Thus, we will include dependent effect sizes in our meta‐analysis. It is likely we will have both between‐study (e.g., same research teams conducting multiple evaluations) and within‐study (e.g., multiple follow‐ups from same study sample) dependency in the meta‐analysis. This dependence structure will inform our chosen meta‐analytical model.

#### 3.6.3 Missing data

Following Lipsey and Wilson (2001), in those reports where key statistical information is missing, we will attempt to obtain data from the corresponding authors of primary studies. When that is not possible, the study will be excluded from the synthesis of effect sizes. All the studies excluded for this reason will be identified and systematically reported. For missing information in relation to other codes (e.g., participant age) we will apply (Pigott & Polanin, 2020) ‘infer, initiate, impute’ guideline for addressing missingness.

#### 3.6.4 Assessing risk of bias in included studies

In line with the original review (Valdebenito et al., 2018) we plan to control the risk of bias in included studies by using Effective Practice and Organisation of Care (EPOC; Cochrane library EPOC, 2017). The instrument proposes the following eight criteria for the assessment of quality bias: (i) sequence generation, (ii) allocation concealment, (iii) baseline outcome equivalence, (iv) baseline characteristics equivalence, (v) incomplete outcome data, (vi) blinding of outcome assessment, (vii) protection against contamination and (viii) selective outcome reporting. Each of these domains will be judged on a 3‐point scale (i.e., low risk, high risk, unclear risk). The EPOC tool provides guidance and examples for each domain that facilitate the decision of assigning low, high or unclear risk. In fact, the EPOC tool proposed here encompasses similar items as those suggested by the Cochrane tool (RoB 2).

Risk of bias of studies involving quasi‐experimental designs will be analysed using the ROBINS‐I, a Cochrane Risk of Bias Assessment Tool for Non‐Randomised Studies of Interventions (Sterne et al., 2016). The ROBINS‐I involves seven domains, namely: (i) bias due to confounding factors, (ii) bias in selection of participants into the study, (iii) bias in classification of interventions, (iv) bias due to departures from intended interventions, (v) bias due to missing data, vi) bias in measurement of outcomes and (vii) bias in selection of the reported results. Each domain includes questions that facilitate the judgement of each single report. Each of these domains would be judged on a 5‐point scale (i.e., yes, probably yes, probably no, no and no‐information risk).

We will report RCT and QED results separately. We will conduct sensitivity analysis for the different levels of bias risk detected.

Risk of bias of qualitative studies will be analysed using the CASP check list (Critical Appraisal Skills Programme, 2013). The tool has been suggested by Cochrane Collaboration and it was specifically developed for qualitative evidence. Using a checklist format, CASP involves ten questions targeting the following domains: (i) clarity of aims and research questions, (ii) congruence between research questions and study design, (iii) recruitment, sampling and data collection, (iv) correct application of methods, (v) richness/conceptual depth of the findings, (vi) exploration of deviant cases and alternative explanations, as well as (vii) reflexivity of the researcher (Noyes et al., 2022). Each question would have a response on a 3‐point scale (i.e., yes, cannot tell and no).

#### 3.6.5 Meta‐analysis

To evaluate the impact of interventions to reduce school exclusions, we will compute a meta‐analysis of effect sizes. This will involve primary (e.g., school exclusion) and secondary outcomes (e.g., antisocial behaviour). As previously discussed, it is likely that we will have both hierarchical (between‐study) and correlated (within‐study) structure of dependency among estimated effect sizes. However, this will only become clear when all effect sizes have been extracted.

RVE will be used to allow for the inclusion of dependent effect sizes in the meta‐analysis. We will use the decision‐tree presented by Pustejovsky and Tipton (2022) to make an informed choice regarding the most appropriate meta‐analytical model.

Meta‐analyses will be computed in *rstudio* using the ‘metafor’ package (Viechtbauer, 2010) and the rscript will be included in the technical appendices of the final report.

#### 3.6.6 Sensitivity analysis

Since meta‐analysis involves a wide range of decisions, we will conduct sensitivity analysis to test the robustness of these decisions (Higgins & Green, 2011). The use of this technique can contribute to increasing the confidence in the pooled effects produced by the analysis. When possible, we will run sensitivity analysis isolating randomised controlled trials and quasi‐experimental designs, distinguishing the role of low/high/unknown risk of bias (i.e., quality of the primary studies), differences between adjusted and unadjusted effect sizes, and the differences between published and unpublished data. In the event of outliers accounting for heterogeneity, we will also re‐run analysis for controlling their presence in the pooled effect sizes calculated. It will be also necessary to run sensitivity analysis on the statistical procedures to compute effect sizes (e.g., transforming effect sizes), and the inclusion of reports presenting missing/incomplete data, among others.

#### 3.6.7 Exploring and assessing heterogeneity

We will report weighted mean effect sizes with robust adjusted standard errors. Heterogeneity between effect sizes is assessed by the *Q*‐value, degrees of freedom and the value of *I* ^2^. We include the *I* ^2^ as the value of *Q* can appear distorted if the number of studies included in a meta‐analysis is small (Higgins, 2003) and it can also be transformed easily to a percentage value.

We will report the variance components for each level of a three level meta‐analytical model and conduct a sensitivity analysis to assess the suitability of the multilevel model.

#### 3.6.8 Moderator analysis

On the condition that we retrieve and include a sufficient number of studies, we will perform analysis to explore the potential role of some specific moderators (covariates) explaining the potential heterogeneity involved in our results. Based on theory and our knowledge of previous research we have anticipated a number of potential effect modifiers that should be extracted from the selected studies and coded on the data collection instrument (Supporting Information: Appendix 1). Those moderators would potentially cover the following aspects.

Participants' demographic characteristics: Previous research suggests that school children from ethnic minorities are more likely to be excluded than Caucasians (e.g., Skiba et al., 2011). Also, boys are over‐represented in exclusion rates when compared with girls (Bowman‐Perrott et al., 2013). We will try to explore the role of ethnicity and gender as moderators of overall effect sizes.

Behavioural problems: Previous findings report that the effect of school‐based prevention programmes can vary depending on pre‐existing behavioural problems (e.g., Ferguson et al., 2007; Lösel & Beelmann, 2006). We plan to explore the role of behavioural problems at moderating overall effect sizes.

Theoretical bases of the interventions: We would be interested in testing whether the theoretical background of interventions (e.g., cognitive‐behavioural, restorative justice) can moderate the effect of intervention in reducing disciplinary exclusion.

Quality of the intervention: Previous research testing the effectiveness of prevention programmes settled in schools demonstrates that well‐implemented programmes—those including training, monitoring and supervision—display better results (e.g., Durlak et al., 2011; Gottfredson & Wilson, 2003; Lösel & Beelmann, 2006).

As previously mentioned, in the event that our data is of an acceptable statistical power we will explore heterogeneity by running meta‐regression. For this purpose we will use the ‘metafor’ package (Viechtbauer, 2010).

In the event that we use moderator analysis involving categorical variables, we anticipate the estimation of models analogous to analysis of variance. If we have at least five studies comparing groups based on categorical variables, we will run analysis under random‐effect model. It is foreseeable that we would use separate estimate of *τ* ^2^ (i.e., variance component) for each group. As has been said before, in our review it is difficult to assume that the true effect between‐studies is the same for all groups. We will follow methodological guidelines provided by Borenstein et al. (2009).

#### 3.6.9 Publication bias

To test publication bias, funnel plots of standard error will be produced. Given that the interpretation of funnel plots can be subjective (e.g., Borenstein et al., 2009), we plan the inclusion of additional statistical tests on the potential publication bias (e.g., Fail Safe N, Trim‐and‐Fill).

### 3.7 Treatment of qualitative research

We will undertake a mixed methods review of interventions to reduce school exclusion, and so our review will include a qualitative evidence synthesis of process evaluations of the included interventions.

Based on recommendations from the Cochrane Handbook (Noyes et al., 2022) we will use the PerSpecTIF framework to inform our inclusion criteria for process evaluations. Thus, our inclusion criteria for process evaluations are as follows.

#### 3.7.1 Perspective

We include process evaluations that present qualitative findings from the perspective of children and young people that participated in an intervention programme to reduce school exclusion. We will also aim to include the perspective of school staff, including teachers, staff, and school leadership if possible.

#### 3.7.2 Setting

We will only include process evaluations of interventions to reduce school exclusions that were implemented in schools in the United Kingdom. Schools and education systems vary across different countries and contexts and thus, the implementations and barriers to implementation will also vary. This review is being used to inform the Youth Endowment Fund Toolkit which was designed to produce evidence about reducing and preventing children and young people from becoming involved in crime and violence in the United Kingdom. It is, therefore, justified to restrict our qualitative evidence synthesis to this setting.

We will discuss any possible differences between process evaluations conducted in the different countries that comprise the United Kingdom (e.g., England, Scotland, Wales, and Northern Ireland).

#### 3.7.3 Phenomenon

We will include process evaluations of interventions to prevent children and young people from being excluded from school. These may have been conducted alongside an impact evaluation (i.e., to measure the quantitative effectiveness) or independently. Both will be included in our qualitative evidence synthesis.

#### 3.7.4 Environment

Similar to the review of impact evaluations, we will include process evaluations of school‐based interventions to prevent school exclusions.

#### 3.7.5 Comparison

We will include process evaluations that included groups that did not participate in an intervention to reduce school exclusion. However, this is not a necessary requirement as we are interested in the barriers and facilitators to implementation of these intervention programmes.

#### 3.7.6 Time

We will only include qualitative data that was collected following the end of an intervention to reduce school exclusion.

#### 3.7.7 Findings

We will only include process evaluations that provide participants' perspectives on the facilitators and barriers to implementation of an intervention. By facilitators, we mean factors that encourage or aid the implementation of an intervention. These may also be factors that participants reported they found were effective or successful in reducing school exclusions. By barrier, we mean factors that hinder or prevent the implementation of the intervention or participants' access to the intervention programme. Supporting Information: Appendix 5 describes the data extraction tool for qualitative evidence.

##### Evidence synthesis

There are many methods for conducting a qualitative evidence synthesis, and the choice of method should be guided by several factors including experience of the team, time and resources available, purpose of the review, audience, and the evidence available (Garside, 2008).

Several pieces of information about our included process evaluations will be recorded using Microsoft Excel. One coder will extract and record information about the intervention (i.e., name and brief description), setting, sample (e.g., age, ethnicity, sex), and methodological design (e.g., semi‐structured interviews, focus groups, mixed‐methods evaluation). Furthermore, the type of analysis used by the process evaluations themselves will be recorded.

We will use a thematic synthesis framework to synthesise information from process evaluations. We will undertake synthesis of qualitative data using a deductive approach, whereby raw information from process evaluations will be recorded, compared, and then grouped into themes and subthemes. We will also record the themes and subthemes identified by primary process evaluations where appropriate.

Similar to its use in primary qualitative research, thematic analysis in qualitative evidence synthesis involves three stages (Thomas & Harden, 2008). First, a ‘line‐by‐line’ approach will be applied to extract information on participants' perspectives and views of the implementation of the intervention. We are particularly interested in perspectives on those factors which participants report were difficult and can be described as a barrier to implementation and factors that participants report were favourable aspects and so could be described as a facilitator to implementation. As the nature of qualitative data often means that full interview transcripts or responses in a focus group are not provided, we will also record the researchers' views and interpretations of their data. Direct quotations from participants will be recorded where possible.

The second and third stages will involve the development of descriptive themes and subthemes respectively (Thomas & Harden, 2008). The information extracted across individual process evaluations will then be synthesised and grouped into themes and subthemes. We will assign codes to these themes and subthemes to be able to distinguish between those themes that relate to barriers and those that relate to facilitators. Often barriers and facilitators may be interlinked or not easily separated and so we will also record this when necessary. An example of our proposed thematic analysis of qualitative data is outlined in Table 3 (Supporting Information: Appendix 4).

The findings from our qualitative evidence synthesis will also be interpreted in conjunction with the findings of the quality assessment using the Critical Appraisal Skills Programme (2013) tool.

## ROLES AND RESPONSIBLIITIES

**Content**: Sara Valdebenito, Darrick Jolliffe.

**Systematic review methods**: Hannah Gaffney and Sara Valdebenito.

**Statistical analysis**: Sara Valdebenito, Hannah Gaffney.

**Qualitative analysis**: Hannah Gaffney and Sydney Hitchcock.

**Information retrieval**: Hannah Gaffney, Sara Valdebenito, Maria Jose Arosemena and Sydney Hitchcock.

## SOURCES OF SUPPORT

This is a funded review by the Youth Endowment Fund.

## DECLARATIONS OF INTEREST

None of the researchers involved in the team present financial interest in this review. None of them have been involved in the development of interventions or systematic reviews on the scope of the present one.

## PRELIMINARY TIMEFRAME

The approximate date for submission of the systematic review is October 2023.

## PLANS FOR UPDATING THE REVIEW

We plan to produce an updated version of the review every 3 years. The lead author will be in charge of coordinating and producing the revised versions.

## Supporting information

Supporting information.

## Footnotes

1 The age bracket is only for reference. We are aware that possible variation would be found across different countries.
