# Supplementary material for: PROTOCOL: School‐based interventions for reducing disciplinary school exclusion: An updated systematic review
Source: Campbell Syst Rev. 2023 Aug 21;19(3):e1344. doi: 10.1002/cl2.1344 (PMC10442604; doi:10.1002/cl2.1344)
Supplement: Supplementary file 1 — Supporting information. [file CL2-19-e1344-s001.docx]

## APPENDIX 1: DATA COLLECTION INSTRUMENT (Quantitative studies)

**DATA-CODING INSTRUMENT**

***School-based interventions for reducing***

***disciplinary school exclusion. A systematic review***

[Variable names in brackets]

Contents

Section A. Codification

Section B. Bibliographical information

Section C. Ethics

Section D. Research design

Section E. Sample

Section F. Primary outcome coding

Section G. Secondary outcomes coding

Section H. Base-line measurements

Section I. Programme delivered

Section J. Follow-up measurement

Section K. Effect sizes

Eligibility checklist

**Section A. Codification**

Instruction: use one data-coding instrument for each manuscript. When more than one manuscript reports the same research project, select one of them as the principal (e.g., the older) and give it an ID number. The following manuscripts should use the same ID but it must be registered in the Crossref field.

**[STUDYID]** Study ID number:

**[CROSSREF1]** Cross reference document identifier:

**[CROSSREF2]** Cross reference document identifier:

**[CROSSREF3]** Cross reference document identifier:

**[DATESCR]** Date of screening:

**[CODER]** Coder Initials:

**Section B. Bibliographical information**

Before completing this section, please be sure that the manuscript is correctly uploaded in the reference manager programme.

**[AUTHOR]** Name of the main author(s):

**[AFFIL]** Main author affiliation:

**[DATEPUB]** Year of publication:

**[DATEFIEDW]** Year of fieldwork (usually reported in a range):

**[COISTATEMENT]** Has the paper included a conflict of interest statement?

- 1. Yes
- 0. No

**[LANGPUB]** Language of the publication:

- 1. English
- 2. German
- 3. Italian
- 4. Spanish
- 5. Portuguese
- 999. Other:________

**[COUNTPUB]** Country of publication:

- 1. UK
- 2. USA
- 3. Canada
- 4. Australia
- 999. Other:_________
- 99. Unknown

**[TYPUB]** Type of publication:

- 1. Journal
- 2. Book/book chapter
- 3. Masters thesis
- 4. PhD/doctoral thesis
- 5. Technical/governmental report
- 6. Conference proceedings
- 999. Other:________

**[AUTDIS]** Main author discipline:

- 1. Education
- 2. Social Work
- 3. Psychology
- 4. Criminal Justice
- 5. Sociology
- 6. Psychiatry/Medicine
- 999. Other:________
- 99. Unknown

**[LOCAT]** How was the study/report located?

- 1. Electronic database
- 2. Web search
- 3. Reference in a book/paper. Please specify:
- 4. Hand search in specialised journal
- 5. Peer/expert suggestion
- 999. Other. Specify:____________

**Section C. Ethics**

**[CONSENT]** Did the study declare the use of “consent agreement forms”?

- 1. Yes
- 0. No
- 999. Other:________
- 99. Unknown

**[SIGNCONS]** Who signed the consent?

- 1. Students
- 2. Parents
- 3. Teachers
- 4. Schools
- 5. Parents and student
- 999. Other. Specify:______________
- 99. Unknown

**Section D. Design**

The present systematic review includes randomised control trials as well as quasi-experimental reports (before/after measure plus a control or comparison group). If the control/comparison group is randomly allocated, non-randomly allocated or matched and no intervention expected to produce impact is provided to it, you will be able to code that group as CONTROL. Subsequently, the TREATMENT group could be understood as the group that receives the intervention, no matter if that condition has been randomly allocated or not.

Please select always the data that is related with the sample effectively analysed.

**[DESTYPE]** What kind of design is this paper based on?

- 1. Randomised controlled trial (true experiment)
- 2. Before-and-after with control/comparison group/s
- 3. Instrumental variable
- 4. Propensity score matching
- 5. Interrupted time series
- 6. Pre/post measures with unmatched control/comparison group
- 7. Inverse probability weighting
- 999. Other. Specify:____________

**[RANDUNIT]** Units of randomization

- 1. Individuals
- 2. Clusters/groups (classroom, schools)
- 999. Other. Specify:____________
- 99. Unknown

**[ANALUNIT]** Unit of analysis

- 1. Students
- 2. Clusters/groups (classroom, schools)
- 999. Other. Specify:____________
- 99. Unknown

**[COMPVAR]** Variables measured to create comparability? (e.g., variables used to match the control and treatment groups)

______________________________________________________________________________________________________________________________

**[MAINSTAT]** What is the main statistical analysis used to produce the final results?

- 1. Multilevel modelling
- 2. Differences of means
- 3. MANOVA
- 4. Chi-squared
- 5. Propensity Score Matching
- 999. Other. Specify:____________

**Section D. Sample**

**[SAMPSELECT]** How was the sample selected?

- 1. Randomly
- 2. Assessment
- 3. Self-selection
- 999. Other. Specify:___________

**[INSAMP]** Initial sample size (i.e., individuals/schools):

**[NUMBFOLL]** Nº of follow-up:

**[FOLLSAMP1]** Follow-up 1 sample size:

**[FOLLSAMP2]** Follow-up 2 sample size:

**[FOLLSAMP3]** Follow-up 3 sample size:

**[NSCHOOL]** Initial number of schools:

**[NSFOLL1]** Follow-up 1 sample size:

**[NSFOLL2]** Follow-up 2 sample size:

**[NSFOLL3]** Follow-up 3 sample size:

**[NCLASS]** Initial number of classes:

**[NCFOLL1]** Follow-up 1 sample size:

**[NCFOLL2]** Follow-up 2 sample size:

**[NCFOLL3]** Follow-up 3 sample size:

Please code here the information on attrition described in the manuscript:

|  | Total number  of students at  Baseline | Total number of students at Follow-up |
| --- | --- | --- |
| Treatment | **[NTREBAS]** | **[NTREFOLL]** |
| Control | **[NCONTBA]** | **[NCONTFOL]** |

**[MEANAGE]** Mean age and standard deviation of overall sample at beginning of intervention:

**[GENDER]** Gender

- % of males
- % of females
- 99. Unknown
- **[LOCAT]** Location of program
- Urban area
- Suburban area
- Rural area
- Mixture of areas
- 99. Not enough information to determine

**[GRADEX]** Grade level of students

- % of students in Elementary school or equivalent
- % of students in Secondary school or equivalent
- % of students in High school or equivalent
- 4. Other:
- 99. Unknown

**[ETHNI]** Predominant ethnicity^[[1]](#footnote-1)^

- 1. % of Caucasian:
- 2. % of Black:
- 3. % of Hispanic:
- 4. % of Asian:
- 5. % of other mixed background:
- 99. Unknown

**[COUNTRY]** Please state the name of the country where schools and sample of students were located when tested.

______________________ (99 if unknown)

**[LUNEX]** Socio-economic status

% of students receiving free/reduced school lunch:

99. Unknown

**[SENEX]** Special Educational Needs

% of students declaring SEN:

99. Unknown

**Section E. Primary Outcome (School Exclusion)**

**[EXCLUSION] Is the manuscript reporting outcomes for school exclusion?**

- 1. Yes
- 0. No

**[TYPEXC]** Type of exclusion measured

- 1. In-school exclusion
- 2. Out-of-school exclusion
- 99. Unknown

**[CHEKTIP]** Duration of school exclusion measured

- 1. Days of Fixed-term exclusion

(Expressed in number or days, frequencies, percentages)

- 2. Days of Permanent exclusion

(Expressed in number or days, frequencies, percentages)

- 99. Unknown

**[ICCEXCLU]** If the statistical analysis include cluster in MLM, please register the ICC for Exclusion:

**Section F. Secondary outcomes^[[2]](#footnote-2)^**

**[BEHAVMES]** Did the study include measures on behaviour domains?

- 1. Yes
- 0. No
- 99. Unknown

What types of the following behaviours are measured?

- **[PROSO]** Pro-social behaviour (e.g., helping, empathy). Specify:____________
- 1. Yes
- 0. No

**[MEPROSO]** Measure(s) used to test the behaviour (name):

**[ALPHAPROSO]**

- Reliability test. Specify alpha value:_____________
- Non reported

| Groups | Effect size before | Effect size after |
| --- | --- | --- |
| Control or comparison | **[PROBC]** | **[PROAC]** |
| Treatment | **[PROBT]** | **[PROAT]** |

**[PAGEPROSO]** Number of the page from where you extract statistical data:

**[ICCPROSO]** If the statistical analysis include cluster in MLM, please register the ICC for behavioural outcomes:

- **[INTERNAL]** Internalising problem behaviour

(e.g., anxiety, depression, attention-deficit and hyperactivity disorder (ADHD), attention deficit, hyperactivity). Specify:____________

- 1. Yes
- 0. No

**[MINTERNAL]** Measure (s) used to test the behaviour (name):

**[ALPHAINTERNAL]**

- Reliability test. Specify alpha value:_____________
- Non reported

| Groups | Effect size before | Effect size after |
| --- | --- | --- |
| Control or comparison | **[PROBC]** | **[PROAC]** |
| Treatment | **[PROBT]** | **[PROAT]** |

**[PAGEINTERNAL]** Number of the page from where you extract statistical data:

**[ICCINTERNAL]** If the statistical analysis include cluster in MLM, please register the ICC for behavioural outcomes:

- **[NAEXTERNAL]** Non-aggressive externalising problem behaviour

(e.g., stealing, lying, graffiti, illegal drugs). Specify: _____________

- 1. Yes
- 0. No

**[MNAEXTERNAL]** Measure used to test the behaviour (name):

**[ALPHANAEXTER]**

- Reliability test. Specify alpha value:_____________
- Non reported

| Groups | Effect size before | Effect size after |
| --- | --- | --- |
| Control or comparison | **[PROBC]** | **[PROAC]** |
| Treatment | **[PROBT]** | **[PROAT]** |

**[PAGENAXTERN]** Number of the page from where you extract statistical data:

**[ICCNAEXT]** If the statistical analysis include cluster in MLM, please register the ICC for behavioural outcomes:

- **[AAGRESEXT]** Aggressive externalising problem behaviour

(e.g., Opposition/defiance, physical aggression, indirect aggression, instrumental aggressions/dominance, reactive aggression, school bullying). Specify:_____________

- 1. Yes
- 0. No

**[MAGRESSEXT]** Measure used to test the behaviour (name):

**[ALPHAAEXT]**

- Reliability test. Specify alpha value:_____________
- Non reported

| Groups | Effect size before | Effect size after |
| --- | --- | --- |
| Control or comparison | **[PROBC]** | **[PROAC]** |
| Treatment | **[PROBT]** | **[PROAT]** |

**[AGRESPAGE]** Number of the page from where you extract statistical data:

**[ICCAGREEX]** If the statistical analysis include cluster in MLM, please register the ICC for Behavioural outcomes:

**Section G. Base-line measurements**

**[DATABAS]** Date of baseline assessment:

What measures were used?

**[SRMES]** Self-report

- 1. Yes
- 0. No
- 99. Unknown

**[TRMES]** Teachers’ report

- 1. Yes
- 0. No
- 99. Unknown

**[SCHRMES]** School records

- 1. Yes
- 0. No
- 99. Unknown

**[PAREP]** Parents

- 1. Yes
- 0. No
- 99. Unknown

**[OMES]** Other:_________________

**[EXCBL]** Frequency of exclusion at baseline (register any measure given by the study)

**Section H. Programme delivered**

This section aims to codify data on the delivery process. Be aware that sometimes final reports do not describe all the data related to delivery. In those cases it would be helpful to search for registered protocols or earlier publications reporting more data on this.

**[PRONAME]** Name of the programme:

**[PROCURRI]** Was the program curricular?

- 1. Yes
- 0. No
- 99. Unknown
- 999. Other. Specify:____________

**[PROEND]** The programme was conducted for:

- 1. Research ends
- 2. Demonstration ends
- 3. Routine
- 99. Unknown
- 999. Other. Specify:_____________

**[PROSIT]** Primary programme site:

- 1. Public school
- 2. Private school
- 3. Other, (specify):_________
- 99. Unknown

**[PROSCH]** Was at least one of the components of the intervention was settled at school?

- 1. Yes
- 0. No
- 99. Unknown

**[PRODEL]** Who delivered the programme?

- 1. External facilitators
- 2. School facilitators
- 3. Both
- 99. Unknown

**[PDBACK]** Deliverer’s background 1

- 1. Social worker
- 2. Psychologist
- 3. Teacher
- 4. Police officers
- 5. Peers
- 999. Other. Specify:_____________
- 99. Unknown

**[PDBACK]** Deliverer’s background 2

- 1. Social worker
- 2. Psychologist
- 3. Teacher
- 4. Police officers
- 5. Peers
- 999. Other. Specify:_____________
- 99. Unknown

**[TRAINBEF]** Did the deliverer receive training BEFORE implementing the programme?

- 1. Yes.
- 0. No.
- 99. Unknown

**[THOURS]** How long was the training in hours?:____________

**[TRAINDUR]** Did the deliverer receive training DURING the implementation?

- 1. Yes.
- 0. No.
- 99. Unknown

**[THOURS2]** How long was the training in hours?:

What type of intervention was delivered? If the manuscript indicates a mixture of interventions you can select more than one using TYPEPRO 1, 2 and 3.

|  | **[TYPEPRO1]** | **[TYPEPRO2]** | **[TYPEPRO3]** |
| --- | --- | --- | --- |
| 1. Mentoring programme |  |  |  |
| 2. Restorative programme |  |  |  |
| 3. Skills training programme |  |  |  |
| 4. School-wide systemic intervention |  |  |  |
| 5. Classroom management |  |  |  |
| 6. Counselling/therapy |  |  |  |
| 999. Other |  |  |  |

Theoretical background of the intervention. If the manuscript indicates a mixture of theories, you can select more than one using THEORY 1, 2 and 3.

|  | **[THEORY1]** | **[THEORY2]** | **[THEORY3]** |
| --- | --- | --- | --- |
| 1. Cognitive behavioural |  |  |  |
| 2. Learning theory |  |  |  |
| 3. Restorative theories |  |  |  |
| 4. Organisational theories or principles |  |  |  |
| 99. Unknown |  |  |  |
| 999. Other (Specify) |  |  |  |

**[PROCONT]** What happened to the control group?

- 1. No intervention
- 2. Wait-list control
- 3. Minimal contact
- 4. Treatment as usual
- 5. Alternative treatment
- 5. Placebo
- 999. Other. Specify:____________

**[PROFORM]** Delivery format:

- 1. Manualised programme
- 2. Unstructured programme
- 3. Mixed
- 99. Unknown
- 999. Other. Specify:____________

What was the programme dosage?

**[PRODOSW]** AVERAGE Duration in weeks:

**[PRODOSH]** AVERAGE Hours per week:

**[PROFREQ]** What was the frequency of the programme counted?

- 1. Less than a week
- 2. Once a week
- 3. Twice a week
- 4. 3-4 times a week
- 5. Daily
- 99. Unknown

**[EVROLE]** What was the “evaluator” role?

- 1. Deliver the programme
- 2. Designed the programme
- 3. Both design and delivery
- 4. Independent evaluator
- 99. Unknown

**[MONITOR]** Was the programme implementation monitored?

- 1. Yes
- 0. No
- 99. Unknown. Not enough information

**[IMPROB]** Does the report provide information about implementation problems?

- 1. Yes, there were clear problems which are reported
- 0. No, non-reported problems, reasonably well implemented
- 2. Possible problems based on the description of the intervention
- 99. Unknown. Not enough information

**[PROCOST]** Is the cost of the intervention mentioned?

- 1. Yes
- 0. No

**[AMOUNT]** Cost:

**[UNITCURR]** Currency:

**Section I. Follow-up measurement**

**[DATEFALL]** Date of follow up:

Multiple follow-ups

**[MONTHFO1]** Nº of months from baseline to 1^st^ follow-up:

**[MONTHFO2]** Nº of months from baseline to 2^nd^ follow-up:

**[MONTHFO3]** Nº of months from baseline to 3^rd^ follow-up:

**[MONTHFO4]** Nº of months from baseline to 4^th^ follow-up:

What measures were used?

**[POSTSR]** Children/adolescent self-report

- 1. Yes
- 0. No

**[POSTTR]** Teachers’ report

- 1. Yes
- 0. No

**[POSTSR]** School records

- 1. Yes
- 0. No

**[POSTPR]** Parents report

- 1. Yes
- 0. No

**[POSTO]** Other:__________

**[FREQEXFOLL]** Frequency of exclusion at follow-up (register any measure given by the study)

**Section J. Effect sizes of intervention on school exclusion**

- Effect size: outcomes expressed in continuous data.

**[CSSEX]** Sample size for the ES (Treatment group)

**[CSSCON]** Sample size for the ES (Control group)

**[MEANEX]** Mean (Treatment group)

**[MEANCON]** Mean (Control group)

**[MEANADJ]** Are the Means adjusted?

- 1. Yes.
- 0. No

**[ADJBY]** Adjusted by (describe):______________

**[SDEX]** Standard deviation (Treatment group)

**[SDCON]** Standard deviation (Control group)

**[SEEX]** Standard error (Treatment group)

**[SECON]** Standard error (Control group)

**[CORREX]** Correlation coefficient + *p* value (Treatment group)

**[CORRCON]** Correlation coefficient + *p* value (Control group)

**[SMDTREAT]** Standardised mean difference + confidence intervals

- Effect size: outcomes expressed in dichotomous data.

**[DSSTRE]** Sample size for the ES (Treatment group)

**[DSSCONT]** Sample size for the ES (Control group)

**[NUMTRE]** Treatment group; number of successful cases:

**[NUMCON]** Control group; number of successful cases:

**[PROPTRE]** Treatment group; proportion of successful cases:

**[PROPCON]** Control group; proportion of successful cases:

**[ORTRE]** Treatment group; odds ratios:

Confidence Intervals:

*p*-value:

**[ORCON]** Control group; odds ratios:

Confidence Intervals:

*p*-value:

**[ORADJ]** Are the odds ratios adjusted?

- 1. Yes.
- 0. No

Adjusted by (explain):_________

**[CHISC]** X^2^ value with *df:*

**[PAGEEFFECT]** Number of the page from where you extract statistical data:

- Effect sizes at follow-up

**[ESFOLLOW1]** Calculated effect at follows up 1:______

[ESFOLL1] Number of months after intervention for follow-up 1:______

**[ESFOLLOW2]** Calculated effect at follows up 2:______

[ESFOLL2] Number of months after intervention for follow-up 2:______

**[ESFOLLOW3]** Calculated effect at follows up 3:______

[ESFOLL3] Number of months after intervention for follow-up 3:______

**[ESFOLLOW4]** Calculated effect at follows up 4:______

[ESFOLL4] Number of months after intervention for follow-up 4:______

## APPENDIX 2: different types of interventions

| **Type of Intervention** | **Targeted population** | **Example** | **Intervention deliverers** |
| --- | --- | --- | --- |
| School-Wide Interventions | School community | School-Wide Positive Behavioural Interventions and Supports (SWPBIS).  The intervention targets a systemic change process in a whole school or in a school district. SWPBIS aims to reduce students’ misbehaviour by i) changing staff approach and ii) developing systems and supports to meet children’s behavioural needs (Bradshaw, Waasdorp, & Leaf, 2012; Noltemeyer, Palmer, James, & Wiechman, 2019). | School staff, teachers, and administrators |
| Classroom management | Teachers | Classroom-centred intervention (CC). Aimed at improving classroom management, the programme involves three main components: i) curriculum enhancement, ii) enhanced behaviour management practices and iii) back-up strategies for children not performing adequately (Ialongo, Poduska, Werthamer, & Kellam, 2001) | Teachers |
| Skills training | School children | Coping Power (CP) attempts to improve a child’s social competence, self-regulation, self-control and social bonds with peers, teachers and caregivers. CP incorporates individual counselling sessions, weekly group meetings and monthly parent meetings (Russell, 2007). | School Psychologists, master’s level clinicians |
| Mentoring programmes | School children | The Rochester Resilience Program involves “In 14 lessons with school-based mentors. During the sessions children are taught a hierarchical set of skills such as monitoring of emotions; self-control/reducing escalation of emotions; and maintaining control and regaining equilibrium. Mentors offer reinforcement of skill use in classrooms” (Wyman et al., 2010, o. 707) | Resilience Mentors |
| Restorative justice and restorative practice. | School community | Two aims guide this approach. First, it attempts to repair harm in case of conflicts and/or incorrect behaviour. Second, restorative practices attempt to build and strengthen relationships, as well as promote and develop relational and personal skills such as empathy, assertiveness and self-efficacy (Lodi, Perrella, Lepri, Scarpa, & Patrizi, 2022). |  |

## APPENDIX 3: SCREENING TOOLS

**Quantitative studies (RCTs and QEDs)**

| **Criteria** | **Evaluation** | |
| --- | --- | --- |
| 1. Does this paper measure school exclusion as an outcome? | - YES | - NO |
| 1. Does the intervention is school based? (or at least one component in the school) | - YES | - NO |
| 1. Are the target individuals school students in mainstream schools? | - YES | - NO |
| 1. Is the report based on an experimental design (RCT)? (if the answer is NO, go to the next item (Item 5, below) | - YES | - NO |
| 1. Is this report based on a QED as per our target?   The study uses before and after measures. Yes/No  The study uses at least one matched control group. Yes/No  The study match treatment and control group using demographics and at least one behavioural risk factor (.e.g., suspensions, violence, disciplinary problems, school absenteeism) Yes/No  The matching procedure produces balance between the treatment and control group Yes/No  **If one of the answers is NO, the study is not our target. Select NO in the last column 🡪 | - YES | - NO |
| Is this report included?  *(If you have selected NO in one or more of the previous questions the study will need to be excluded)* | - YES | - NO |
| Reasons for exclusion: | | |

**Qualitative studies**

| **Criteria (PerSpecTIF)** | **Evaluation** | |
| --- | --- | --- |
| *Perspective: (i.e., the sample)*  Does the paper present findings from the perspective of children and young people, or school staff/teachers/school leaders? | - Yes | - No |
| *Setting:*  Does the paper present findings from a UK study? | - Yes | - No |
| *Phenomenon:*  Does the paper present findings of a process evaluation of an intervention? | - Yes | - No |
| *Environment:*  Does the paper present findings with school students in mainstream schools? | - Yes | - No |
| *Time:*  Does the process evaluation present findings at the end of an intervention or during an intervention? | - Yes | - No |
| *Findings:*  Does the report present findings on school exclusion/suspension? | \| - Yes \|  \| \| --- \| --- \| | - No |
| **Is this paper included?** | \| - Yes \|  \| \| --- \| --- \| | - No |
| Reasons for exclusion: | | |

## APPENDIX 3: Example of electronic search

ERIC via ProQuest

14/02/2023

| MAINSUBJECT.EXACT("Suspension") OR MAINSUBJECT.EXACT("Zero Tolerance Policy") OR MAINSUBJECT.EXACT("Expulsion") |
| --- |
| TI,AB(expuls* OR expel* OR suspen* OR "stand down") |
| 1 or 2 |
| MAINSUBJECT.EXACT("Schools") OR MAINSUBJECT.EXACT("Middle Schools") OR MAINSUBJECT.EXACT("Private Schools") OR MAINSUBJECT.EXACT("Junior High Schools") OR MAINSUBJECT.EXACT("High Schools") OR MAINSUBJECT.EXACT("Public Schools") OR MAINSUBJECT.EXACT("Elementary Schools") OR MAINSUBJECT.EXACT("Secondary Schools") |
| MAINSUBJECT.EXACT("High School Students") OR MAINSUBJECT.EXACT("Junior High School Students") OR MAINSUBJECT.EXACT("Middle School Students") OR MAINSUBJECT.EXACT("Secondary School Students") OR MAINSUBJECT.EXACT("Elementary School Students") |
| TI,AB(school or schools) |
| TI,AB((school* OR elementary OR primary OR "middle school*" OR "junior high" OR secondary OR "high school*" OR grade*) NEAR/4 (student* OR children)) |
| 4 or 5 or 6 or 7 |
| TI,AB(intervention* OR program* OR prevention* OR policy OR policies OR initiative* OR strateg*) |
| MAINSUBJECT.EXACT("Mixed Methods Research") OR MAINSUBJECT.EXACT("Randomized Controlled Trials") OR MAINSUBJECT.EXACT("Evaluation Research") OR MAINSUBJECT.EXACT("Qualitative Research") |
| TI,AB("randomi* control trial*" or rct or experiment* or "quasi experiment*" or impact* or effect* or efficacy or efficien* or evaluation* or assess* or qualitative or implement* or process*) |
| 10 or 11 |
| 3 and 8 and 9 and 12 |

## APPENDIX 4: DATA COLLECTION INSTRUMENT (Quanlitative studies)

Table 3

*Example of coding framework for process evaluations of interventions to reduce school exclusions.*

|  | **Description** | **Example** |
| --- | --- | --- |
| **Study ID** | A study identifier assigned by coder to process evaluation. | Study_1 |
| **Author(s)** | Surname of first author | Jolliffe et al. |
| **Year** | Year of publication | 2022 |
| **Intervention name** | Name of intervention if provided. | School-Wide Positive Behavioral Support or restorative justice |
| **Intervention description** | Brief description of the intervention components, activities, and/or aims | A whole-school intervention that involved classroom projects and teacher training to promote a positive school climate, improve classroom management strategies, and reduce disruptive behaviour. |
| **Location of evaluation** | Where the intervention was implemented | London (Brixton) |
| **Sample** | Information about the study sample from whom qualitative data was collected. | 10 secondary school teachers of which 4 were identified as male and 20 secondary school pupils of which 13 were identified as male. No information about ethnicity or age of participants. |
| **Design/Methodologies** | How the qualitative data was collected | Semi-structured interview |
| **Analysis** | Type of analytical approach used to interpret and report data | Thematic analysis |
| **Findings Theme** | Description of themes identified in process evaluations | Lack of time and resources |
| **Theme: evidence** | Evidence associated with the theme. | Direct quotations and extracts from process evaluations |
| **Subthemes** | Description of subthemes identified in process evaluation | Teacher availability  Balancing priorities |
| **Subtheme: evidence** | Evidence associated with the subtheme. | Direct quotations and extracts from process evaluations |
| **Code** | Barrier or facilitator to implementation | Barrier |

*Note.* Examples are hypothetical and do not reflect any real process evaluation of an intervention to reduce school exclusion.

## APPENDIX 5: DATA COLLECTION INSTRUMENT (Quanlitative studies)

Table 3

*Example of coding framework for process evaluations of interventions to reduce school exclusions.*

|  | **Description** | **Example** |
| --- | --- | --- |
| **Study ID** | A study identifier assigned by coder to process evaluation. | Study_1 |
| **Author(s)** | Surname of first author | Jolliffe et al. |
| **Year** | Year of publication | 2022 |
| **Intervention name** | Name of intervention if provided. | School-Wide Positive Behavioral Support or restorative justice |
| **Intervention description** | Brief description of the intervention components, activities, and/or aims | A whole-school intervention that involved classroom projects and teacher training to promote a positive school climate, improve classroom management strategies, and reduce disruptive behaviour. |
| **Location of evaluation** | Where the intervention was implemented | London (Brixton) |
| **Sample** | Information about the study sample from whom qualitative data was collected. | 10 secondary school teachers of which 4 were identified as male and 20 secondary school pupils of which 13 were identified as male. No information about ethnicity or age of participants. |
| **Design/Methodologies** | How the qualitative data was collected | Semi-structured interview |
| **Analysis** | Type of analytical approach used to interpret and report data | Thematic analysis |
| **Findings Theme** | Description of themes identified in process evaluations | Lack of time and resources |
| **Theme: evidence** | Evidence associated with the theme. | Direct quotations and extracts from process evaluations |
| **Subthemes** | Description of subthemes identified in process evaluation | Teacher availability  Balancing priorities |
| **Subtheme: evidence** | Evidence associated with the subtheme. | Direct quotations and extracts from process evaluations |
| **Code** | Barrier or facilitator to implementation | Barrier |

*Note.* Examples are hypothetical and do not reflect any real process evaluation of an intervention to reduce school exclusion.

1. Based on Lipsey & Wilson (2001) [↑](#footnote-ref-1)
2. Opposition/defiance, physical aggression, indirect aggression, instrumental aggressions/dominance, reactive aggression, school bullying, antisocial behaviour or cime, are expected to be captured in the variable **AAGRESEXT.** [↑](#footnote-ref-2)
